# Melatonin Alleviates Radiation-Induced Lung Injury via Regulation of miR-30e/NLRP3 Axis

**DOI:** 10.1155/2019/4087298

**Published:** 2019-01-10

**Authors:** Xu Wu, Haiying Ji, Yuli Wang, Chenlin Gu, Wenyu Gu, Lijuan Hu, Lei Zhu

**Affiliations:** ^1^Department of Pulmonary Medicine, Zhongshan Hospital, Fudan University, Shanghai 200032, China; ^2^Department of Pulmonary Medicine, The First Affiliated Hospital, College of Medicine, Zhejiang University, Hangzhou 310003, China; ^3^Department of Urology, Shanghai Tenth People's Hospital, Tongji University School of Medicine, No. 301, Yanchang Rd., Shanghai 200072, China

## Abstract

Melatonin is a well-known anti-inflammatory and antioxidant molecule, which plays a crucial role in various physiological functions. In this study, mice received a single dose of 15 Gy radiation delivered to the lungs and daily intraperitoneal administration of melatonin. After 7 days, mice were processed to harvest either bronchoalveolar lavage fluid for cytokine assays or lungs for flow cytometry and histopathological studies. Herein, we showed that melatonin markedly alleviated the oxidative stress and injury, especially suppressing the infiltration of macrophages (CD11b+CD11c−) and neutrophils (CD11b+Ly6G+) to the irradiated lungs. Moreover, in the irradiated RAW 264.7 cells, melatonin blocked the NLRP3 inflammasome activation accompanied with the inhibition of the IL-1*β* release and caspase-1 activity. However, melatonin restored the downregulated miR-30e levels. Quantitative PCR analysis of miR-30e and NLRP3 indicated the negative correlation between them. Notably, immunofluorescence staining showed that overexpression of miR-30e dramatically diminished the increased NLRP3 expression. Luciferase reporter assay confirmed that NLRP3 was a target gene of miR-30e. Western blotting revealed that transfection with miR-30e mimics markedly reduced the expressions of NLRP3 and cleaved caspase-1, whereas this phenomenon was reversed by the miR-30e inhibitor. Consistent with this, the beneficial effect of melatonin under irradiated exposure was blunted in cells transfected with anti-miR-30e. Collectively, our results demonstrate that the NLRP3 inflammasome contributed to the pathogenesis of radiation-induced lung injury. Meanwhile, melatonin exerted its protective effect through negatively regulating the NLRP3 inflammasome in macrophages. The melatonin-mediated miR-30e/NLRP3 signaling may provide novel therapeutic targets for radiation-induced injury.

## 1. Introduction

Radiation is part of the radiotherapy for thoracic malignancies, such as nonsmall cell lung cancer (NSCLC). Conventionally fractionated radiotherapy for NSCLC consists of 1.8–2.0 Gy fractions given once daily to a total dose of 60 Gy or more [1]. Currently, available treatment strategies are challenged by the adverse effects of radiation in normal tissues surrounding the tumors, which preclude the application of curative radiation doses [2]. Radiation-induced lung injury is a kind of sterile inflammatory response to ionizing radiation, which occurs in three phases, namely, acute inflammatory phase, intermediate healing phase, and late fibrotic phase. Remarkably, radiation pneumonitis is an increasingly prevalent cause of morbidity and mortality [3]. The precise mechanisms underlying this pathology may involve radiation damage to the alveolar epithelium and capillary endothelium or an innate immune response to tissue inflammation [4, 5]. Besides, radiation helps to drive a persistent ROS generation in lung tissue, which could be produced by a variety of cell types. The nucleotide-binding domain-like receptor protein 3 (NLRP3) inflammasome is a cytosolic multiprotein complex of the innate immune system, consisting of the NOD-like receptor (NLR) NLRP3 scaffold, the adaptor protein ASC (apoptosis-associated speck-like protein containing a caspase recruitment domain), and pro-caspase-1 [6]. Accumulation of ROS is a major trigger of NLRP3 inflammasome activation, which promotes the cleavage of caspase-1 and the maturation of proinflammatory cytokines such as IL-1*β* [6, 7]. A previous study provided new insight into the NLRP3 inflammasome activation in radiation-induced tissue damage [7, 8]. However, the potential role of NLRP3 inflammasome in radiation-induced lung injury will have to be clarified.

Melatonin (N-acetyl-5-methoxytryptamine) is secreted by the pineal gland in mammals and many other organs. The biological functions of melatonin involve a wide spectrum of pathophysiological processes, including circadian rhythm [9], inflammation [10], oxidative stress [11], and metabolism [12]. In particular, melatonin is able to scavenge free radicals directly or through its stimulatory actions on antioxidant enzyme activity, as well as through inhibitions on prooxidative enzyme activity [13]. Based on the fact that oxidative stress and inflammatory infiltration participate in radiation-associated injury, we develop a proposal that melatonin might serve as a therapeutic agent to mitigate the tissue damage. Emerging studies have well established that inflammasome was a novel molecular target for melatonin [14]. They have extensively demonstrated that melatonin could inhibit the NLRP3 inflammasome pathway in radiation-induced oral mucositis [15] and cecal ligation-induced sepsis [16]. Nevertheless, the underlying mechanism on how melatonin mediates NLRP3 inflammasome is largely unclear.

MicroRNAs (miRs) have been proven to act as key regulators of the cellular machinery and physiological processes, especially in the modulation of NLRP3 inflammasome [17]. Recently, it has been suggested that melatonin significantly enhanced the expression of microRNAs [18], which bind to specific complementary sequences in the 3′-untranslated regions (UTRs) of target mRNAs and control the posttranscriptional regulation [19]. Nevertheless, the correlation between melatonin and miRNA/NLRP3 signaling caused by radiation challenge still remains elusive. In this study, using cultured cells and a mouse pulmonary radiation model, we evaluated the effect of melatonin on the oxidative stress activities (as index of lipid peroxidation) and inflammatory injury in targeted lung tissues exposed to *γ*-rays. We also explored the novel miRNA mechanism and cellular immune mediators involved in the radioprotection. The improved mechanistic understanding of radiation-induced pneumonitis is a prerequisite for the development of more effective radiotherapy.

## 2. Material and Methods

### 2.1. Animal Model and Lung Irradiation Procedure

Male C57BL/6 mice (6–7 weeks old, 22–24 g) were purchased from Shilek Lab Animal (Shanghai, China). All animals were maintained under standard conditions (22 ± 2°C, 55 ± 5% humidity, and 12 h light/dark cycle) with regular chow and water. The mice were acclimatized for 1 week. Radiation was performed on anesthetized mice (intraperitoneal pentobarbital sodium 50 mg/kg). Mice were placed in a specifically designed, well-ventilated plastic box and given a single dose (15 Gy) of *γ*-rays at a dose rate of 1.35 Gy/min to the whole thorax. The irradiation source was ^60^Co from the radiation center (Nuclear Medicine Institute of Fudan University, China).

To investigate the protective effects of melatonin against radiation-induced lung injury, the WT mice were randomly divided into control (nonirradiated) + saline vehicle, irradiated + saline vehicle, and irradiated + intraperitoneal injection of melatonin groups, with the melatonin group serving as the control for intraperitoneal melatonin vehicles. Melatonin (M5250, Sigma-Aldrich, St. Louis, MO, USA) was freshly dissolved in 1% ethanol (in normal saline) as a stock to produce 10 mg/mL working dilutions in saline. Mice were intraperitoneally injected with 1 mg/day melatonin or the same volume of saline for 7 days post irradiation. This experiment was performed using 40 mice with ten in each of the four groups. After radiation, animals were housed (four or five animals per cage) and maintained in the university's facility. All mice were monitored daily and euthanized 7 days after the last dose of melatonin. When animals were sacrificed, lung tissues and bronchoalveolar lavage fluid (BALF) were harvested and processed appropriately for the different analyses. The BALF was harvested via injection and retraction of 1 mL PBS-EDTA three times. The collected BALF was then centrifuged. The cell-free supernatant was aliquoted and stored at −80°C in preparation of cytokine detection. All experimental procedures were approved by the Animal Care Committee of Fudan University and were performed according to the Guidelines of Laboratory Animal Care and Use.

### 2.2. Cell Culture and Transfection

The human tracheobronchial epithelial cell line BEAS2-B was obtained from the Cell Research Center for Biomedical Research (Institute of Development, Aging and Cancer, Tohoku University) and was grown in culture dishes or culture slides with maintenance medium containing 10% FBS and 1% penicillin-streptomycin in a 5% CO_2_ humidified chamber. The RAW 264.7 cells (originating from the Chinese Academy of Medical Science, Shanghai, China) were cultured in DMEM medium supplemented with 10% fetal bovine serum (FBS), 100 U/mL of penicillin, 100 U/mL of streptomycin, and 3 mM glutamine at 37°C in a humidified atmosphere containing 5% CO_2_. In all experiments, cells were allowed to acclimate in serum-free culture medium for 24 h before 15 Gy irradiation. The microRNA mimics (miR-30e mimics), miRNA mimic negative control (miR-NC), and miRNA inhibitors for miR-30e were purchased from GenePharma (Shanghai, China). The miR-30e mimic was synthesized with the following sequence: sense, 5′-UGUAAACAUCCUUGACUGGAAG-3′ and anti-sense, 5′-UCCAG UCAAGGAUGUUUACAUU-3′. The transient transfection assays were performed using Lipofectamine 2000 according to the manufacturer's protocol. After 48 h transfection, the RAW 264.7 cells were exposed to 15 Gy irradiation and then treatment with melatonin (500 *μ*mol/L) for 12 h. Finally, the cells were used for luciferase assay or western blotting analysis.

### 2.3. Histopathological Evaluation

Half of the lung samples were fixed in 10% buffered formalin for 48 h and embedded in paraffin. Multiple sections (4 *μ*m thick) were deparaffinized with xylene and stained with hematoxylin and eosin (H&E) for pathological analysis. Trichrome blue was used to stain collagens in lung tissue sections.

### 2.4. Determination of Pulmonary Oxidative Stress Production

The lung tissues were homogenized in lysis buffer and centrifuged at 10,000 ×g for 10 minutes at 4°C. The supernatants were collected to detect the malondialdehyde (MDA) content and activities of superoxide dismutase (SOD) using commercial kits (Beyotime, China) following the manufacturer's instructions. All results were normalized to the protein concentration and expressed as U/mg protein or nmol/mg protein as appropriate. The intracellular reactive oxygen species (ROS) in RAW 264.7 cells was detected by determining the oxidation of DCFH-DA. After irradiation treatment, the cells were incubated with DCFH-DA at 37°C in a dark place for 30 min.

### 2.5. ELISA for Cytokines

The lung homogenates and the culture supernatants were collected, and the levels of IL-1*β* and IL-18 were measured using mouse enzyme-linked immunosorbent assay (ELISA) kits (R&D System, MN, USA) according to the manufacturer's instructions.

### 2.6. Flow Cytometry

Lung digests were obtained by incubation with 1 mg/mL collagenase A and 100 ng/mL DNase (Sigma-Aldrich) for 2 h. The cell debris was removed by a cell strainer (BD Pharmingen, San Jose, CA). Red blood cells were removed by red blood cell lysis buffer (eBioscience, San Diego, CA). Cells (0.5–1 × 106) from lung digests were stained with antibodies including BV421-conjugated anti-F4/80, APC-conjugated anti-CD206, PerCP-cy5.5-conjugated anti-Ly6C, BV480-conjugated anti-CD11c, PE-Cy7-conjugated anti-Ly6G, PE-conjugated anti-CD45, and FITC-conjugated anti-CD11b (eBioscience). Analysis was performed on a FACScan cytometer (Becton Dickinson, Mountain View, CA). The FAM-FLICA caspase-1 detection kit (ImmunoChemistry Technologies LLC, Bloomington, MN, USA) was used to detect activated caspase-1 of RAW 264.7 cells according to the manufacturer's protocol. All data were analyzed on FlowJo software (Tree Star Inc., San Carlos, CA).

### 2.7. Quantitative Reverse Transcription (qRT-PCR)

For miR-30e quantitative analysis, total RNA was isolated using the miRNeasy isolation kit (Qiagen); 2 *μ*g of retrieved total RNA was reversely transcribed using stem-loop antisense primer mix and AMV transcriptase (TaKaRa, China) according to the manufacturer's instructions. Extraction of total RNA of RAW 264.7 cells using a TRIzol reagent (Invitrogen) was performed as described. Quantitative assay of NLRP3 expressions was performed using a SYBR QPCR kit (Toyobo, Osaka, Japan). Real-time PCR was routinely performed on an ABI 7500 real-time PCR system (Applied Biosystems, CA, USA). The primer sequences were as follows: for miR-30e, sense: 5′-ACACTCCAGCTGGGTGTAAACATCCTTGAC-3′ and antisense: 5′-CTCAACTGGTGTCGTGGAGTCGGCAATTCAGTTGAGCTTCCA-3′; for U6, sense: 5′-CTCGCTTCGGCAGCACA-3′ and antisense: 5′-A ACGCTTCACGAATTTGCGT-3′. Finally, the relative amounts of miR-30e to U6 and NLRP3 to GAPDH were calculated using the 2^−∆∆Ct^ method.

### 2.8. Immunofluorescent Staining

The cells were washed with PBS, permeabilized for 10 min with 0.1% Triton X-100 in PBS, washed with PBS, and incubated with primary antibodies of NLRP3 overnight at 4°C, as previously described [17]. After samples were washed with PBS, the secondary Alexa Fluor® 488-conjugated antibody (Santa Cruz) was added and incubated for 1 h at room temperature. The nuclei were counterstained with DAPI (Vector Laboratories). Images were obtained by using an Olympus DP70 digital camera and software (Olympus), with constant camera exposure settings throughout.

### 2.9. Luciferase Reporter Assay

NLRP3 3′UTRs containing conserved miR-30e binding sites as well as the mutated sites were synthesized by GenePharma and amplified by qPCR. The cells were cotransfected with either wild-type or mutant NLRP3 3′UTR and 100 ng reporter plasmid, plus miR-30e mimic/inhibitor or control for 48 h. Luciferase activity was measured using a Dual-Luciferase Kit (Promega) and expressed as relative to the activity of the negative control.

### 2.10. Western Blot Analysis

40 *μ*g of proteins was transferred onto a PVDF membrane following separation on a 10% SDS-polyacrylamide gel. The membrane was blocked with blocking solution (5% (*w*/*v*) nonfat dry milk) for 1 h, followed by an overnight incubation at 4°C with a specific primary antibody. Membranes were probed with antibodies against NLRP3, ASC, pro-caspase-1, cleaved caspase-1, and *β*-actin (Santa Cruz Biotechnology, Santa Cruz, CA, USA) according to previously described methods. The following day, the membrane was incubated for an additional 1 h with HRP-conjugated secondary antibody (1 : 5000 dilution) at room temperature after thoroughly washing three times with PBST. Bands were detected by ECL (Amersham Pharmacia Biotech Inc., Piscataway, NJ) and quantified using ImageJ gel analysis software. All experiments were performed in triplicate.

### 2.11. Statistical Analysis

All statistical analyses were conducted with SPSS 19.0 software. Correlations between miR-30e and NLRP3 expressions in lung tissues were assessed with the Pearson correlation coefficient. *p* < 0.05 was considered significant.

## 3. Results

### 3.1. Melatonin Protects against Radiation-Induced Lung Damage and Oxidative Stress

**Figure 1:**
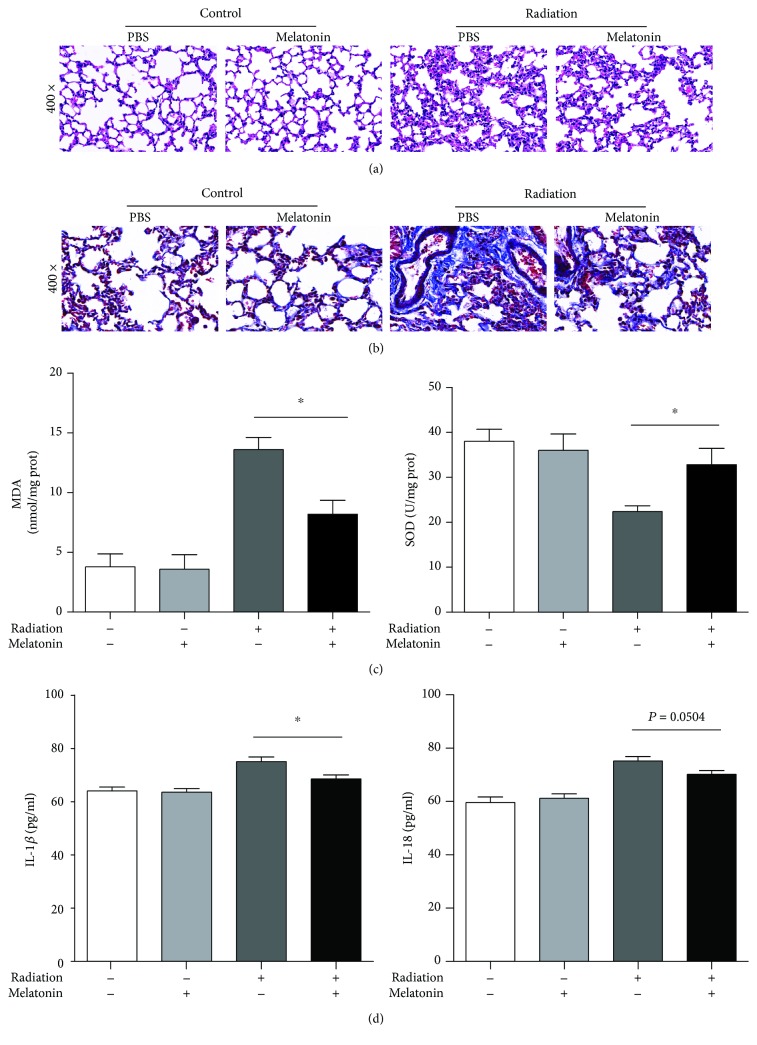
Melatonin protects against radiation-induced pulmonary damage and oxidative stress. (a) Morphological observations obtained from the control, radiation, radiation + melatonin (i.p.), and melatonin groups. Representative light microscopy photographs of HE are shown (magnified 400x). (b) Masson's trichrome staining of the lung (indicates collagen deposition). (c) Effects of melatonin on levels of MDA and SOD from lung homogenates. (d) Melatonin attenuated cytokine expression in the lung. Levels of cytokine secretion of IL-1*β* and IL-18 in BALF were measured by ELISA. All data are presented as means ± SEM (*n* = 6 in each group). ^∗^ *p* < 0.05 vs. the radiation group.

The histopathological effects of acute radiation-induced pneumonitis in the lungs are characterized by edema, exudation, alveolar septal thickening with mononuclear cell infiltration, vascular congestion, etc. Similarly, by means of histopathological analysis, we confirmed that radiation caused a striking infiltration of leukocytes and swelling of the alveolar interstitium reflective of pneumonitis in mice, but irradiated mice treated with melatonin demonstrated less damage, as evidenced by areas of thinner alveolar septa and less infiltration of leukocytes (Figure 1(a)). Masson trichrome staining revealed extensive collagen accumulation in the lung of mice after radiation, but irradiated mice treated with melatonin had mild fibrotic changes in alveolar areas (Figure 1(b)). The oxidative stress marker including superoxide dismutase activity and malondialdehyde level indicated that the generation of reactive oxygen species (ROS) increased in response to radiation. In our study, melatonin remarkably reduced the MDA content (8.2 ± 2.59 nmol/mg prot) and restored the SOD level (32.8 ± 8.17 U/mg prot) in the irradiated lung tissues. IL-1*β* and IL-18 are the main products of the NLRP3 inflammasome activation. Measurements of the IL-1*β* levels in bronchoalveolar lavage fluid showed that radiation increased the inflammatory cytokines significantly, compared with either radiation + melatonin (75.14 ± 4.29 vs. 68.61 ± 3.68 pg/mL; *p* < 0.01, Figure 1(d)) or control (64.12 ± 3.5 pg/mL; *p* < 0.01). However, in contrast to the radiation group, treatment of melatonin after radiation did not significantly change the IL-18 levels in BAL fluid (75.19 ± 4.13 vs. 70.22 ± 3.45 pg/mL; *p* > 0.05). All these findings indicate that melatonin treatment can reduce irradiation-induced oxidative stress and suppress acute damage and subsequent fibrotic damage of the lungs after irradiation.

### 3.2. Melatonin Modulates Macrophage Subsets and Neutrophils in Response to Radiation

**Figure 2:**
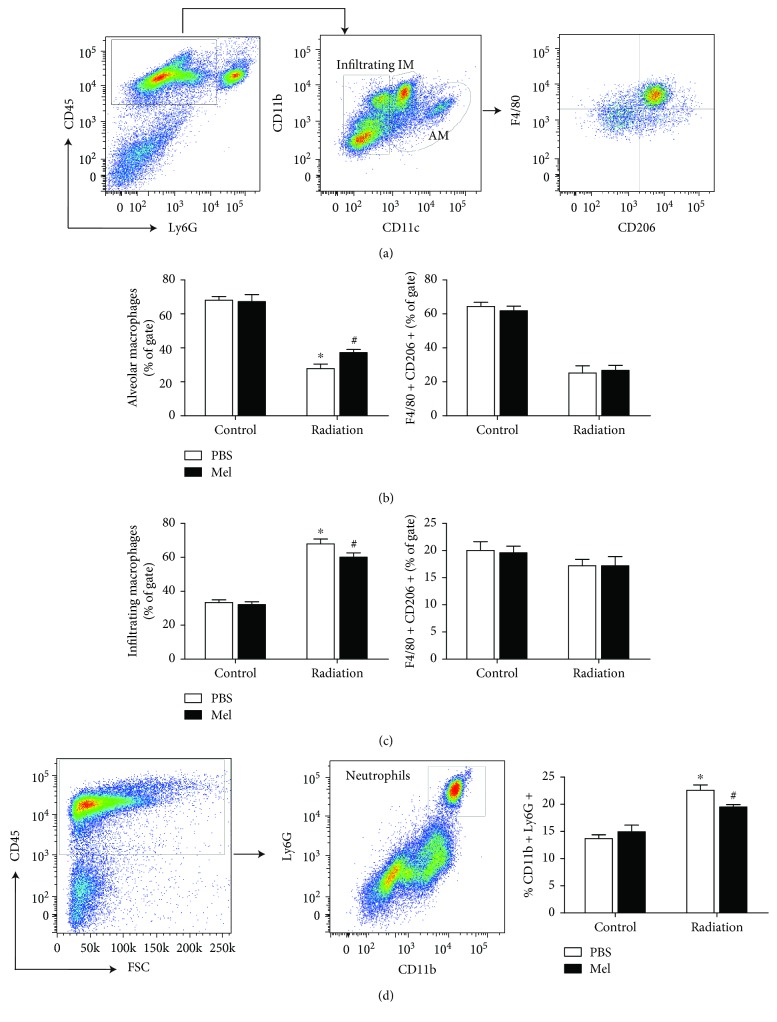
Representative flow cytometry plots of lung single-cell suspensions from the control, melatonin, and radiation-treated mice. (a) The gating strategy of CD45+ myeloid lung cells for characterization of pulmonary populations, such as infiltrating macrophages (CD11b+CD11c−) and alveolar macrophages (CD11b int CD11c+). Percentages of F4/80+CD206+ AM macrophage subsets (b) and F4/80+CD206+ infiltrating subsets (c) were statistically analyzed. (d) Percentages of CD11b+Ly6G+ neutrophils within CD45+ cells in the lungs were statistically analyzed. Means ± SEM are shown (*n* = 6 in each group). ^∗^ *p* < 0.01 compared with the control group. ^#^ *p* < 0.05 compared with the radiation group. Mel, melatonin; IM, interstitial macrophage; AM, alveolar macrophage.

Macrophages are recruited as a first response to radiation-induced damage and regarded as the primary source of inflammatory cytokines [20]. Alterations in macrophages following radiation have been observed during the early phase of tissue injury. Macrophages were gated based on differential expression of CD11b and CD11c to analyze the infiltrating macrophages (CD11b+CD11c−), interstitial macrophages (IM), and alveolar macrophages (AM, CD11b int CD11c+) (Figure 2(a)). Irradiated mice tended to have a lower number of alveolar macrophage subsets compared with control (27.74 ± 6.11% vs. 68 ± 5.15%), while supplementation with melatonin seemed to protect this population (37.2 ± 4.15%; *p* < 0.05; Figure 2(b)). In contrast, our flow cytometry analysis of lung parenchyma subsets of residual F4/80+CD206+ macrophages in irradiated lung tissue did not show a difference compared with the radiation + melatonin group (*p* > 0.05; Figures 2(b) and 2(c)). As an immunoregulatory role, the percentages of CD11b+CD11c− infiltrating macrophage subsets were markedly induced by radiation compared with control (67.92 ± 6.43% vs. 33.34 ± 3.62%; *p* < 0.05); however, after treatment of melatonin, there was a decreased percentage of infiltrating macrophages post irradiation (60.12 ± 5.54%; *p* < 0.05).

Neutrophils were gated by the expression of CD11b and Ly6G/Gr-1 markers within the CD45+ population (Figure 2(d)). The percentage of CD11b+Ly6G+ neutrophils in the lungs was significantly increased after radiation compared with the control (22.57 ± 1.75% vs. 13.67 ± 1.24%). In addition, supplementation of melatonin also prevented the influx of activated neutrophils in the lung caused by radiation (19.5 ± 0.79%; Figure 2(d)). These findings suggest that the modulation of macrophage subset functions and neutrophil infiltration in response to radiation may play a critical role in melatonin-mediated radioprotective effects in the lungs.

### 3.3. Melatonin Suppresses Radiation-Induced NLRP3 Inflammasome Activation in RAW 264.7 Cells

**Figure 3:**
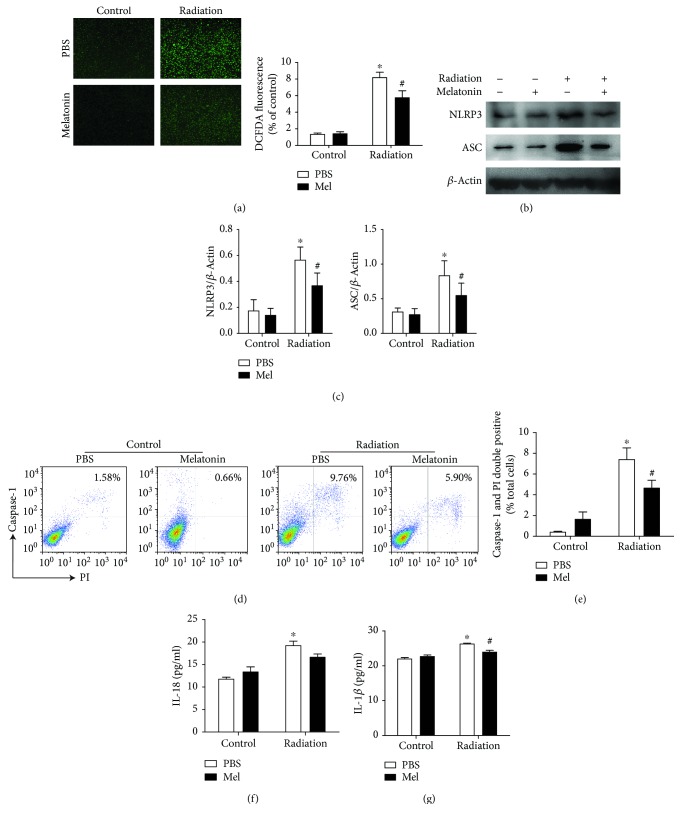
Melatonin suppresses radiation-induced NLRP3 inflammasome activation in RAW 264.7 cells. The RAW 264.7 cells were subjected to 15 Gy *γ* radiation and then treated with melatonin (500 *μ*mol/L) for another 12 h. (a) Effect of melatonin on radiation-triggered ROS production in RAW 264.7 cells. The level of ROS was measured by intracellular fluorogenic probe staining (green). Higher intensity of fluorescence represented more ROS generation (magnification is ×200). *n* = 3 per group. (b) Radiation exposure significantly induced NLRP3 inflammasome activation. Protein expressions of NLRP3 and ASC were detected by western blot analysis. (c) Quantification of relative protein expression was performed by densitometric analysis, and *β*-actin acted as an internal control. Similar results were obtained from three independent experiments. (d) The representative images of activated caspase-1 and PI double-positive proportions in RAW 264.7 cells were measured by flow cytometry. (e) The quantitative histograms from the obtained results. Levels of IL-18 (f) and IL-1*β* (g) in culture supernatants were measured by ELISA. All data are presented as the means ± SEM. ^∗^ *p* < 0.01 vs. the control group. ^#^ *p* < 0.05 vs. the radiation group. *n* = 6 per group. Mel, melatonin.

Regarding to the effect of melatonin on the radiation-induced immune response in vivo, we further investigated the molecular mechanism of melatonin-mediated macrophage response in vitro. Since melatonin is a well-known scavenger of free radicals, we then determined the oxidant production. In the current study, the content of intracellular ROS was significantly increased in the RAW 264.7 cells after exposure to 15 Gy *γ* radiation relative to controls. Concurrently, we also observed a less cellular ROS production in RAW 264.7 cells treated with melatonin, compared with radiation alone (*p* < 0.001, Figure 3(a)), which indicated that melatonin can effectively suppress the production of a ray-generated large amount of reactive oxygen substances within the target cells.

Western blot analysis showed that the expressions of NLRP3 and ASC significantly increased following 15 Gy irradiation, whereas melatonin treatment markedly attenuated the NLRP3 and ASC activations compared with those vehicle-treated (Figures 3(b) and 3(c)). Likewise, as shown in Figures 3(d) and 3(e), the proportion of double-positive cells (activated caspase-1 and PI) analyzed with flow cytometry was obviously increased after radiation (7.39 ± 2.27%), while the caspase-1^+^PI^+^ cells were significantly attenuated by melatonin (4.65 ± 1.54%, *p* < 0.05). Notably, similar results were obtained with ELISA assays in the supernatant of a cell culture medium. The secretion of IL-1*β* and IL-18 exposed to 15 Gy radiation were dramatically elevated (Figures 3(f) and 3(g)). Nevertheless, melatonin did not have a significant effect on the production of IL-18. Taken together, these data indicate that melatonin could block radiation-induced NLRP3 activation and IL-1*β* production in macrophages.

### 3.4. Melatonin Mediates miR-30e/NLRP3 Inflammasome Signaling

**Figure 4:**
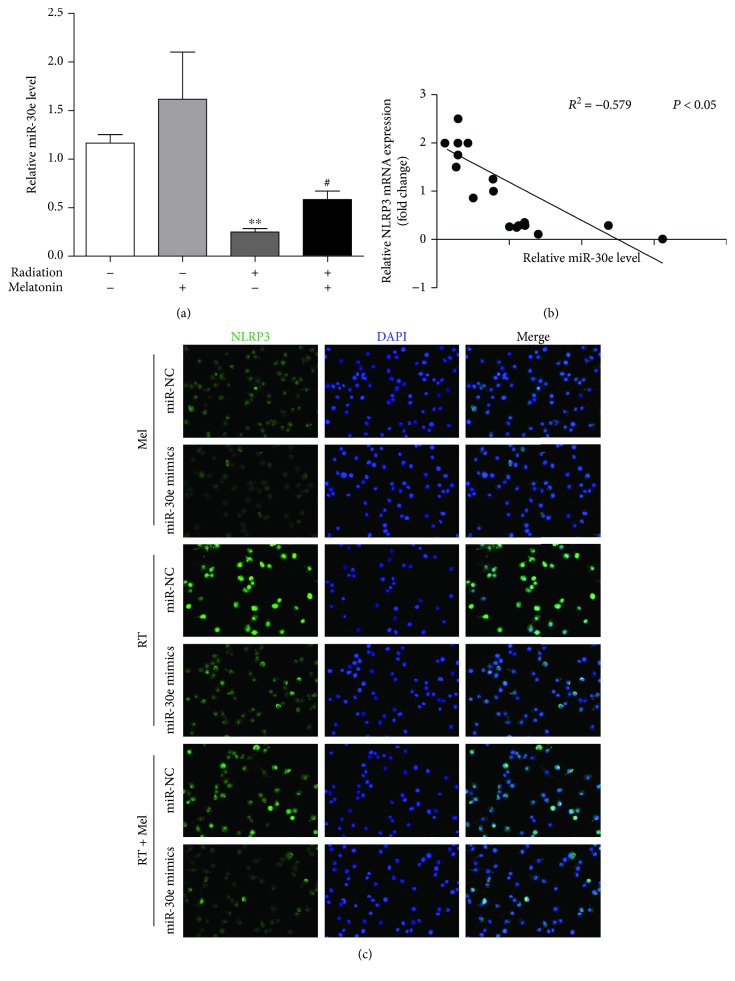
Melatonin mediates the miR-30e/NLRP3 inflammasome axis. (a) Effect of melatonin on the expression of miR-30e following radiation exposure in RAW 264.7 cells. The relative quantification of miR-30e-3p was normalized using the U6 results. *n* = 4 per group. (b) RT-qPCR analysis was performed to investigate the correlation between miR-30e and NLRP3 mRNA levels for individual samples. The data are presented as the means ± SEM. ^∗∗^ *p* < 0.01 vs. the control group. ^#^ *p* < 0.05 vs. the radiation group. (c) Representative images of immunofluorescence staining with NLRP3 (green) (magnification is ×400) in the cytoplasm of irradiated RAW 264.7 cells are shown. The robust NLRP3 expression was reversed by melatonin treatment (500 *μ*mol/L), while it was dramatically blunted by the miR-30e mimic after radiation challenge. Cell nuclei were stained with DAPI (blue). Similar results were obtained from three independent experiments. RT, radiation; Mel, melatonin.

The posttranscriptional regulation by microRNAs (miRs) such as the translational repression or mRNA degradation is critical in lung injury-associated pathogenesis. Li et al. reported that miR-30e directly targeted NLRP3 in microglial cells using a miRNA target gene prediction website (http://www.microrna.org/) [21]. As a result, we analyzed the interaction between miR-30e and NLRP3 following radiation. Interestingly, quantitative PCR analysis of miR-30e showed that, although radiation increased miR-30e levels, administration with melatonin restored miR-30e to ∼2-fold (Figure 4(a)). Whenever exposed to radiation, there existed a negative correlation between the expressions of NLRP3 and miR-30e (*R* ^2^ = −0.579, *p* < 0.001, Figure 4(b)). Thus, we speculated that the rescued miR-30e levels by melatonin could suppress the NLRP3 inflammasome. To study this possibility, we examined the endogenous NLRP3 inflammasome activity under conditions in which we enhanced miR-30e expression using the miR-30e mimic. Consistently, radiation-treated cells displayed a striking NLRP3 immunofluorescence staining that was detected in the cytoplasm, as shown in Figure 4(c). However, the irradiated cells transfected with si-NC showed more robust NLRP3 staining than those with the miR-30e mimic. Moreover, in collaboration with the miR-30e mimic, melatonin remarkably reduced NLRP3 expression (Figure 4(c)).

**Figure 5:**
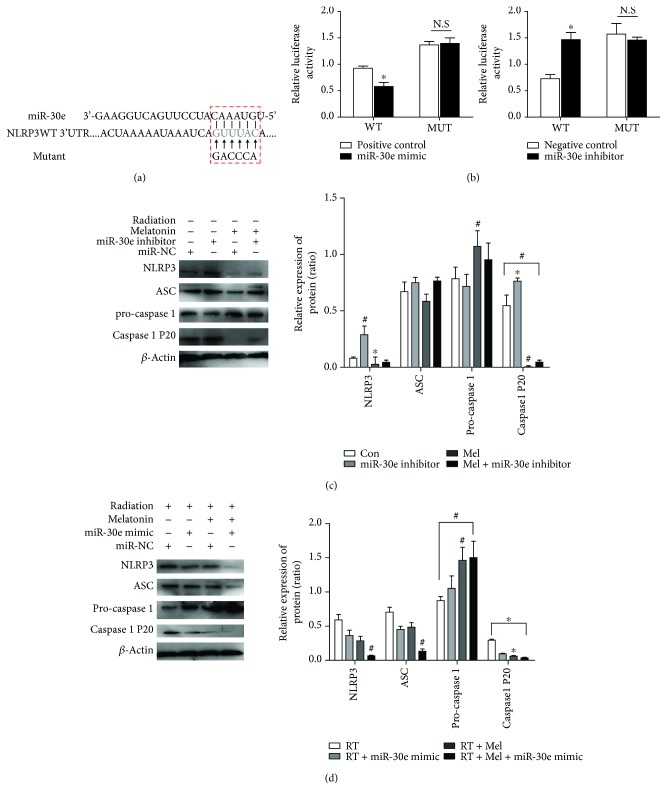
miR-30e/NLRP3 inflammasome signaling is involved in the melatonin-mediated radioprotection in RAW 264.7 cells. (a) A predicted miR-30e binding site in the 3′UTR of NLRP3 and the complementary wild-type NLRP3 sequence or mutant sequences are shown. (b) The luciferase assay was performed to verify if NLRP3 is a target gene of miR-30e. The cells were cotransfected with either miR-30e mimics or the miR-30e inhibitor or their corresponding negative control (miR-NC) and either wild-type (WT) or mutant NLRP3 3′UTR. Data are expressed as the means ± SEM. ^∗^ *p* < 0.05 vs. mimic negative control or inhibitor negative control, *n* = 3; N.S, not significant. (c) The beneficial effect of melatonin on the elevated NLRP3, ASC, and cleaved caspase-1 (p20) expressions was negated by a miR-30e inhibitor. The data are presented as the means ± SEM. ^∗^ *p* < 0.05 vs. the control group. ^#^ *p* < 0.01 vs. the control group. (d) Transfection of miR-30e mimics markedly decreased NLRP3 protein expression. The amounts of each protein were quantified by densitometry and expressed relative to the amount of *β*-actin in the same samples. The data are presented as the means ± SEM. ^∗^ *p* < 0.05 vs. the RT group. ^#^ *p* < 0.01 vs. the RT group. RT, radiation; Mel, melatonin.

As displayed in Figure 5(a), a 6 bp fragment of the 3′UTR of the NLRP3 gene is complementary to the miR-30e seed sequence. To verify this prediction, we inserted the 3′UTR sequence of NLRP3 into the luciferase reporter construct. As illustrated in Figure 5(b), the miR-30e mimic markedly suppressed the luciferase activity to 62% compared to the si-NC (scramble sequence of miR-30e) group, in cells transfected with a wild-type NLRP3 3′UTR reporter, but not in cells transfected with the mutated one. In addition, cotransfection of the mutated NLRP3 3′UTR with an miR-30e inhibitor markedly increased the luciferase activity to 49%. However, miR-30e had no effect on luciferase activities elicited by the mutations.

**Figure 6:**
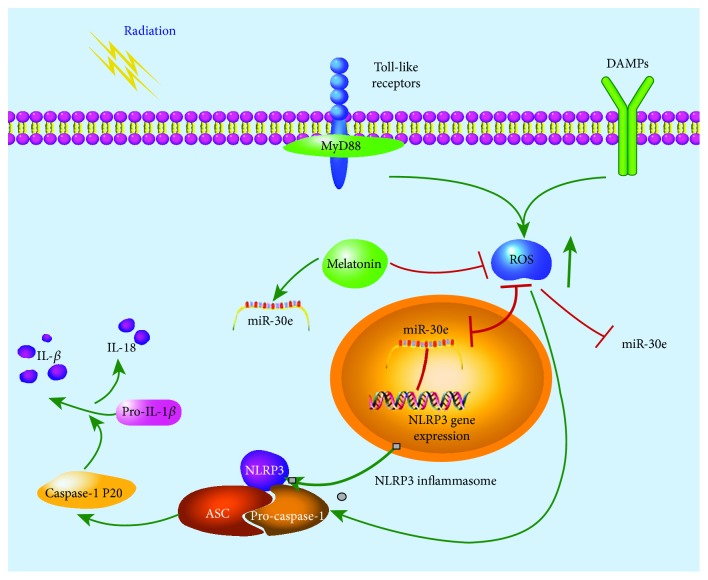
A schematic model illustrating the underlying mechanism of the alleviated effect of melatonin on radiation-induced lung injury through the miR-30e/NLRP3 signaling pathway. TLRs and DAMPs activated by radiation further promoted the ROS generation and, secondly, the transcription of NLRP3. Moreover, miR-30e was downregulated in the macrophages of an irradiated cell model but its target gene NLRP3 was upregulated, thus leading to the enhanced assembly of the NLRP3 inflammasome, then the cleavage of pro-caspase-1 and pro-IL-1*β*. Green lines: facilitation. Red lines: inhibition.

Although the immunostaining clearly reflected the NLRP3 inflammasome activation, we further performed immunoblotting to analyze the two forms partitioned by caspase-1, including the precursor of caspase-1 (pro-caspase-1, p45) and mature cleaved caspase-1 (p20). From Figures 5(c) and 5(d), we detected that protein expressions of NLRP3 and cleaved caspase-1 were significantly upregulated upon radiation stimulation except for ASC, whereas the upregulations were partially abolished by melatonin, respectively. In contrast, inhibition of miR-30e with its inhibitor markedly rescued the activated NLRP3 inflammasome in irradiated RAW 264.7 cells, while the protective effect of melatonin was dramatically attenuated in the anti-miR-30e-transfected groups. Of note, the alteration of cleaved caspase-1 was inversely associated with the expression of pro-caspase-1 in cells transfected with the miR-30e inhibitor or mimics. Overall, these results suggest that melatonin exerted its protective effect on radiation-induced injury through negatively regulating the miR-30e/NLRP3 axis in macrophages, as illustrated in Figure 6.

## 4. Discussion

Here, we demonstrate for the first time that melatonin counteracts the radiation-induced direct oxidative stress damage and manipulates immune response by affecting macrophage subsets, thus contributing to its radioprotection. In detail, melatonin abrogated the radiation-induced formation of infiltrating macrophage clusters accompanied by phenotypic alternation. Of note, we have revealed that radiation-induced NLRP3 inflammasome activation was attenuated by melatonin exposure, while this effect of melatonin was markedly negated by repressing miR-30e levels with its inhibitor in macrophages. These results support the hypothesis that NLRP3 inflammasome activation contributes to the pathogenesis of radiation-induced lung injury. Therefore, targeting the NLRP3 inflammasome by melatonin may represent a novel strategy to limit the radiation-induced loss of immune cells, cascades of proinflammatory cytokines, and related tissue damage.

Radiation-induced acute lung injury develops a complex pathological process involving acute inflammation, increased collagen production, and ROS generation. More noteworthy, overproduction of ROS in multiple cellular environments can specifically affect the sensitive modifying enzymes of the redox system and repair proteins that play a pivotal role in both early and late effects of radiation. Fardid et al. have shown that preadministration of melatonin could overcome the indirect destructive effect of radiation by modulating cyclooxygenase-2 (COX-2) and inducible NO synthase (iNOS) levels in out-of-field lung tissue after partial body irradiation [22]. In the present work, melatonin directly prevented histological damage to the irradiated lung and cultured epithelial cells, possessing the properties against the progression of inflammatory injury. Similar results were obtained with Zhang et al., who found that the IP injection of melatonin markedly reduced the pulmonary injury and decreased the infiltration of macrophages and neutrophils into the lung [23]. As reported by others [24], melatonin protects the irradiated cells by supporting endogenous antioxidant enzymes such as superoxide dismutase and glutathione peroxidase, while suppressing prooxidant enzymes. Accordingly, the radioprotection mechanism can be attributed to the scavenging of free radicals and attenuation of lipid membrane peroxidation and neutrophil-induced infiltration [25].

In particular, activated macrophages lead to iNOS production which contributes to COX-2 induction. As a consequence, lung tissue with plenty of macrophages is vulnerable to the radiation and the macrophages are probably the main sources of ROS in an irradiated lung. In an attempt to prove the assumption, we confirmed that melatonin inhibited the generation of ROS in the cultured macrophages. Regardless, macrophages are regarded as the primary source of proinflammatory cytokines, thus initiating the increased cytokine cascade greatly. Consistent with Groves et al.'s study [26], the percentage of pulmonary CD11b int CD11c+ alveolar macrophages declined during the first week in irradiated mice, which was paralleled by an increased influx of CD11b+Ly6G+ neutrophils. It is well known that this myeloid cell population contains macrophage precursors [27]. Our data indicated that an increase in the CD11b+CD11c+ interstitial macrophages was elicited by radiation. As suggested by others [28], we therefore speculate that the subsequent increase in monocyte chemoattractants induced by radiation could trigger the influx of myeloid cells into the lung tissue to replace the depleted tissue-resident macrophages, thus prompting the radiation-induced tissue damage. Additionally, exposure to radiation can influence these peripheral immune cells, which in turn impair the recovery of destroyed cells and even result in a systemic response syndrome. In response to microenvironmental signals, macrophages could become phenotypically polarized. Surprisingly, our data show that radiation did not result in a significant loss of the proportion of cells that expressed F4/80+CD206+. Therefore, modulation of the immune cells in response to radiation at least partially, correlated with a mechanism of radioprotection [29].

It is evident that radiation increases cytokines and chemokines in mice [30]. IL-1*β* and IL-18 have shown significant increases in a radiation dose-dependent manner [31]. Herein, the inflammatory changes were accompanied by the increased IL-1*β* and IL-18 levels. Moreover, we found that melatonin significantly reduced the level of IL-1*β* in the BALF. Consistent with our results, another study reported that melatonin downregulated IL-1*β* production in LPS-stimulated RAW 264.7 macrophages [32]. It has been reported that the maturation and release of large quantities of IL-1*β* require the activated caspase-1. Our data showed that treatment with melatonin greatly reduces the NLRP3 inflammasome activity and cleaved caspase-1 p20 in the irradiated macrophages. Cao et al. also found that melatonin treatment significantly alleviated liver injury by suppressing the TXNIP-NLRP3 inflammasome pathway [33]. The exact mechanism of NLRP3 inflammasome activation remains elusive. It is believed that mitochondrial ROS are the key factors that are required for NLRP3 activation [34]. Ionizing radiation induces the generation of ROS and reactive nitrogen species (RNS). As the common NLRP3 inflammasome trigger, ROS/RNS could activate the inflammation cascade that drives acute and chronic inflammation [35]. Indeed, pharmacological or RNAi-based inhibition of NADPH oxidase could suppress the NLRP3 inflammasome activation. Previous investigations implicate that administration of the antioxidant dramatically abolished ROS production, thereby reducing the levels of NLRP3 [36]. Thus, redox mechanisms regulate transcription of IL-1*β* and NLPR3 as well as IL-1*β* secretion and caspase-1 activity.

Moreover, excessive production of ROS caused MAPK dephosphorylation, which could be activated by multiple exogenous stimuli such as ultraviolet or ionizing radiation [37]. In the previous work, we blocked p38 MAPK signaling through the inhibitor SB203580 and found that it significantly suppressed the expression of the NLRP3 inflammasome and IL-1*β* and cleavage of caspase-1 [38]. MAPK signaling might represent a potential role in NLRP3 inflammasome activation in irradiated lung tissue. However, we did not examine the effects of melatonin on radiation-stimulated activation of p38 MAPK, JNK, and ERK by measuring their phosphorylated forms. It is known that exposure directly to radiation or ROS will result in both nucleus and mitochondria DNA damages. Further, cell death after radiation occurs by mitotic catastrophe and by apoptosis [39]. In addition, activation of MAPK is closely correlated with the induction of the mitochondrial apoptotic pathway. Of note, the activated macrophages undergo caspase-1-dependent cell death without the characteristic oligonucleosomal fragmentation pattern associated with apoptosis. Unlike caspase-9 (35 and 37 kDa) or active caspase-3 (17 and 19 kDa) caspase-dependent signaling cascades, caspase-1-dependent cell death is associated with potassium efflux, cell swelling, release of proinflammatory intracellular contents, and formation of pores (1-2 nm) in the plasma membrane, which are positive for propidium iodide (PI) staining [40]. In the supplementary data (available here), we clearly detected the intense PI staining in tracheobronchial epithelial cells post irradiation. Whether ablating caspase 1 activity in vitro could rescue the damage after radiation remains unclear. Nevertheless, there lacks direct evidence with regard to the inhibitors of apoptosis as intrinsic regulators of the caspase cascade and having a crucial role on miR-30e or NLRP3 axis. The primary cultured lung cells are considered to be the best experimental model for in vitro studies. However, it is relatively difficult to isolate primary cultured lung cells due to their limited proliferation activity. The RAW 264.7 as a model of a macrophage is also supported by findings in other related studies of the NLRP3 inflammasome [41]. Furthermore, melatonin reduced the level of radiation-induced apoptosis in tracheobronchial epithelial cells. Based on these observations, we proved our hypothesis that melatonin exerted its protective effect on radiation-induced injury through negatively regulating the miR-30e/NLRP3 axis in macrophages.

Several groups have observed that miR-30e reduction led to multifactorial inflammatory changes including hyperactive NF-*κ*B, IL-1*β*, and TNF-*α* [42, 43]. Likewise, in an irradiated cell model, our findings are in agreement with their observations that reduced miR-30e following radiation was associated with the increased inflammatory responses. In contrast, Li et al. reported that knockdown of NF-*κ*B-p65 significantly suppresses radiation-induced miR-30 expression, while suppression of miR-30 protects mice and human CD34+ cells from radiation injury [44]. Interestingly, Beer et al. tried to select the differentially expressed miRNAs in the irradiated human PBMCs 20 h after high-dose radiation [45]. In accordance with our data, they indeed found a significant downregulation of miR-30e in a silico analysis. Our in vitro study showed that, similar to the effect of melatonin, miR-30e mimics strikingly diminished the radiation-induced NLRP3 expression as evidenced by the immunofluorescent staining. As expected, our immunoblotting analysis further confirmed that the elevated miR-30e expression induced by melatonin significantly suppressed theNLRP3 pathway, whereas the beneficial effect of melatonin was negated by a miR-30e inhibitor. These findings established a link between melatonin and the miR-30e/NLRP3 inflammasome pathway in radiation-induced acute lung injury. Although some other miRNAs have been identified to be upregulated in melatonin-treated cells [46, 47], the exact mechanism of how melatonin directly regulates miR-30e in normal tissues needs to be further warranted.

## 5. Conclusions

Taken together, we provide the first evidence that as a potential antioxidant, melatonin could effectively ameliorate radiation-induced lung injury by targeting the miR-30e/NLRP3 inflammasome. Hence, knowledge about the rationale for chronic oxidative damage in affected cells and organs following radiation exposure may facilitate the development of treatment strategies for complications associated with radiotherapy.

## Data Availability

The data used to support the findings of this study are included in the article.

## Supplementary Materials

Supplementary Materials Melatonin reduced the level of apoptosis induced by irradiation using the irradiated tracheobronchial epithelial cells BEAS2-B as models of lung damage in vitro shown in the Supplementary data.
