# Supplementary material for: Melatonin Alleviates Radiation-Induced Lung Injury via Regulation of miR-30e/NLRP3 Axis
Source: Oxid Med Cell Longev. 2019 Jan 10;2019:4087298. doi: 10.1155/2019/4087298 (PMC6348879; doi:10.1155/2019/4087298)

Supplementary data

Melatonin reduced the level of apoptosis induced by irradiation using the irradiated tracheobronchial epithelial cells BEAS2-B as models of lung damage in vitro.

(A) Cell death after radiation exposure was measured by propidium iodide (PI, dead cells) or Hoechst 33342 (nuclei) staining (magnification is ×400). (B) Quantitation of propidium iodide/Hoechst fraction showed that melatonin effectively suppressed irradiation induced-cell death to levels that similar to those controls. Data were presented as mean ± SEM (n = 4). *P<0.01 as compared with Radiation group.

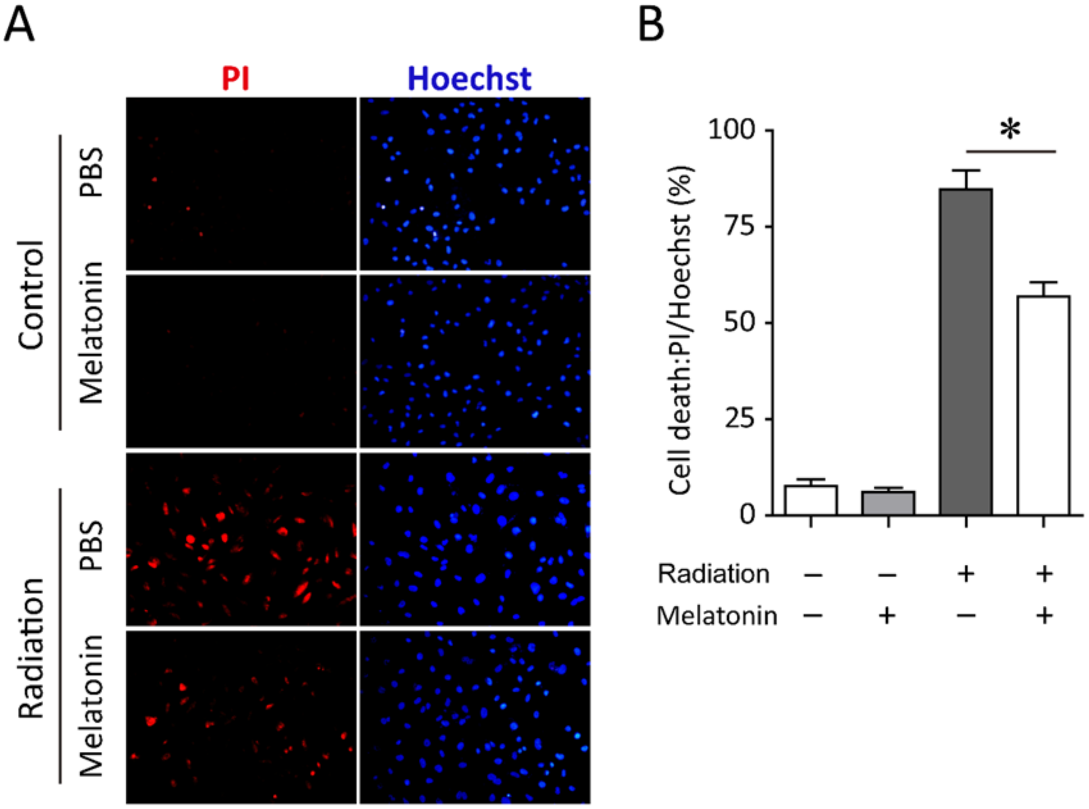

Supplement: Supplementary Materials — Melatonin reduced the level of apoptosis induced by irradiation using the irradiated tracheobronchial epithelial cells BEAS2-B as models of lung damage in vitro shown in the Supplementary data. [file 4087298.f1.pdf]
